# Editorial: Haemorrhoidal Disease: Old Solutions and Future Perspectives

**DOI:** 10.3389/fsurg.2022.905570

**Published:** 2022-04-12

**Authors:** Sara Z. Kuiper, Gaetano Gallo, Mario Trompetto, Arcangelo Picciariello, Stéphanie O. Breukink

**Affiliations:** ^1^ Department of Surgery, School of Nutrition and Translational Research in Metabolism (NUTRIM), Maastricht University, Maastricht, Netherlands; ^2^ Department of Medicine, Surgery and Neurosciences, Unit of General Surgery and Surgical Oncology, University of Siena, Siena, Italy; ^3^ Department of Surgery, Clinica Santa Rita, Vercelli, Italy; ^4^ Department of Emergency and Organ Transplantation, University of Bari Aldo Moro, Bari, Italy; ^5^ Department of Surgery, School for Oncology and Reproduction (GROW), Maastricht University, Maastricht, Netherlands; ^6^ Department of Surgery, Maastricht University Medical Centre, Maastricht, Netherlands

**Keywords:** haemorrhoidal disease, office-based procedures, conservative treatment, haemorrhoids, surgical treatment

**Editorial on the Research Topic**

***Haemorrhoidal Disease: Old Solutions and Future Perspectives***

Throughout our history haemorrhoidal disease (HD) has troubled humanity. HD is one of the best-described diseases in medical documentation and records date back to ancient Egyptian and Mesopotamian times (1, 2). A ground for the abundance of antique references on HD could be the high prevalence of this disease, which has not changed over time. About one third of the population is affected by HD (3). Despite the widespread occurrence and an impressive amount of scientific research, the full picture of HD has not yet been grasped. This special issue aims to highlight several dynamically evolving domains in current HD research, ranging from historical viewpoints to technical solutions and patient involvement.

As we can read in the extensive historical overview by Pata et al., the surgical management of HD has altered over the past centuries. With the introduction of anaesthetics and antisepsis in the 19th century, surgery transformed from a butchering art to a modern science, broaching a whole new world of opportunities. Taking away the agony for an awake patient during an operation, most people were initially content with the possibility of performing surgery under general or epidural anaesthesia. However, nowadays, more and more studies show that there is a broad support base for local anaesthesia in HD operations. Colleagues Poskus et al. underline this view, showing local perianal anaesthetic infiltration to be safe and effective for anorectal surgery, with fewer postoperative complications and a reduction of costs. Likewise, Tomasicchio et al. state that a Milligan-Morgan haemorrhoidectomy performed under local anaesthesia and in an outpatient setting is not only successful but has a high patient satisfaction rate as well.

The treatment of HD is based on the severity of prolapse according to the Goligher grading, even if the latter is much debated due to the inappropriate consideration of the patient's symptoms and quality of life (4–6). For low grades, a stepwise approach is advised by Tutino et al., starting with sclerotherapy and – in case of relapse – rubber band ligation (RBL). Indeed, in recent years there has been a rise in the use of the former while rubber band is still the most common office-based procedure (7, 8).

For higher grades of HD, Giordano and Schembari describe a modification of the mucopexy and haemorrhoidal dearterialization by adding an anolift to address the prolapsing component in HD. Pietroletti et al. studied the efficacy of a new formulation in rectal cream, containing Zn-L-Carnosine, in relieving acute symptoms of HD. Zinc-L-Carnosine is a cytoprotective compound stimulating mucosal repair in the gastrointestinal tract and shows to be a safe and effective treatment for bleeding or thrombosed haemorrhoids.

Eberspacher et al. focus more on the process after the operation and introduce self-mechanical anal dilatation as a simple trick to minimize postoperative pain and stenosis after haemorrhoidectomy with radiofrequency. The same author presents an in-depth analysis of the wall layers included in the stapled rectal ring of mucosectomies. In this article, Eberspacher et al. demonstrate that a mucosectomy entails a resection of the full rectal wall and that a “full-thickness” resection does not correlate with a higher rate of post-operative complications.

In line with the first treatment step for all grades of haemorrhoids, described in the European international guideline for HD (9), it is of paramount importance to optimize a patient’s lifestyle and to indicate risk factors. In the review by De Marco and Tiso, the authors stress the intake of adequate fluids, regular exercise, improving anal hygiene, and avoiding straining at stool.

Additionally, the authors mention the importance of good communication between the doctor and the patient, emphasizing on skilful listening. Understanding the disease-burden of a patient can assist in creating the best treatment approach. The usage of a patient-reported outcome (PROM) can be a valuable tool in this matter. Kuiper et al. evaluate the different PROMs available in the field of HD and endorse the use of such a tool in clinical practice to optimize personalized HD treatment (10, 11).

This special issue of *Frontiers in Surgery* on haemorrhoids addresses several topics including non-surgical solutions, technical operative aspects, and the involvement of patient’s experiences with HD. Despite the high incidence of this disabling disease, we know that the level of evidence of treatment remains low. To overcome this issue, a Core Outcome Set (COS) for HD can be utilized in clinical research (12, 13).

By using a COS, which is a minimal set of outcomes, study results can be easier compared to one another. Furthermore, the patient’s view should also be taken into account to ensure a patient tailored approach in the management of HD.

We encourage authors in the field of HD to continue their research to stimulate further discussion and understanding of haemorrhoids.
